# Changes in Arsenic, Copper, Iron, Manganese, and Zinc Levels Resulting from the Application of Poultry Litter to Agricultural Soils

**DOI:** 10.3390/toxics6020028

**Published:** 2018-05-14

**Authors:** Richard D. Foust, Michael Phillips, Killian Hull, Dariia Yehorova

**Affiliations:** 1 Department of Chemistry and Biochemistry, James Madison University, Harrisonburg, VA 22807, USA; killian.hull@gmail.com (K.H.); yehorodx@dukes.jmu.edu (D.Y.); 2 Natural Resources Conservation Service, Virginia, United States Department of Agriculture, Harrisonburg, VA 22801, USA; mike.phillips@va.usda.gov

**Keywords:** poultry litter, soil, iron, manganese, copper, zinc, arsenic, *Cynodon dactylon*

## Abstract

Twelve applications of poultry litter were made to a 2.1-ha field located in the Shenandoah Valley of Virginia, United States (USA), between March 1999 and August 2014. The field was planted with bermudagrass (*Cynodon dactylon*) and used as a pasture on an active farm. Copper, iron, manganese, zinc, and arsenic concentrations in the poultry litter were measured, and the application rates of these metals were calculated. The median application rates were: Cu, 1.32 kg/ha, Fe, 5.57 kg/ha, Mn, 1.80 kg/ha, Zn, 1.39 kg/ha, and As, 0.011 kg/ha. Twelve surface and subsurface soil samples were taken from the treated field in February 2016. Twelve samples were also taken from a comparison site. The comparison site was directly adjacent to the study site, consisted of the same soil type, and had been maintained as an undisturbed forest. Extractable Cu, Fe, Mn, Zn, and As concentrations in the soil samples were determined by atomic absorption spectroscopy, and the results of the chemical analysis were analyzed by ANOVA. Fe and Mn were depleted from the soil in the treated field, while Cu and Zn levels increased over the 12 years of treatment and grazing, and arsenic levels were unchanged in both the surface and subsurface soils between the comparison and the study site. The changes observed for Cu, Fe, Mn, and Zn are within the critical deficiency level and critical toxicity level for these metals, and no arsenic remains in the soil from roxarsone feed supplements, which were added to the poultry feed when the litter was applied to the study site.

## 1. Introduction

The global production of chicken broiler meat in 2017 was 90,175,000 metric tons. The United States is the largest producer of chicken broiler meat, producing 18,690,000 metric tons of chicken broiler meat in 2017 [1]. Poultry production tends to be concentrated in small regional areas. In the United States, three states (Georgia, Alabama, and Arkansas) raised over 3,400,000,000 broiler chickens in 2016, which was 39% of the total production for the United States [2]. Virginia ranks 10th in the United States in the production of broiler chickens and sixth in the production of turkeys. Most of the poultry production in Virginia is located in the Shenandoah River Valley [2].

**Figure 1:**
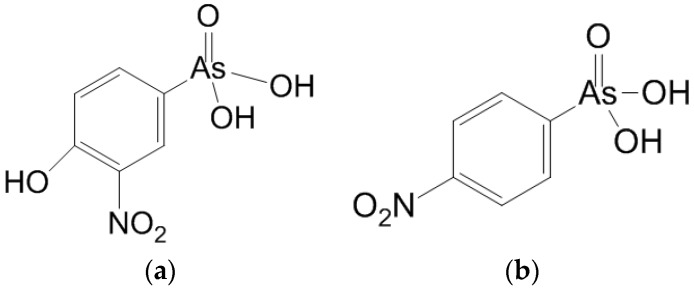
Roxarsone, 4-hydroxy-3-nitrobenzenearsonic acid (**a**), and, nitarsone, (4-nitrophenyl) arsonic acid (**b**). Roxarsone and nitarsone are the two arsenic-based substances that are added to poultry feed that is used in the Shenandoah Valley.

Roxarsone, the commercial name for 4-hydroxy-3-nitrobenzenearsonic acid (see Figure 1a), was approved for use as an animal feed supplement 21 March 1944 [3]. Roxarsone was initially added to poultry feed to prevent coccidiosis, which is a parasitic disease that attacked poultry [4]. It was later approved as a feed supplement for increased weight gain and improved pigmentation in chickens [5]. Nitarsone, (4-nitrophenyl) arsonic acid (see Figure 1b), is a close analog of roxarsone and was approved for the prevention of blackhead disease (histomoniasis) in poultry. Nitarsone was primarily used in turkey feed, where blackhead disease is a significant cause of mortality [5].

A 2004 report by Lasky et al. [6] demonstrated that the As concentration in chicken livers was higher than previously thought, and suggested that a portion of the As in feed is retained by chickens, and may threaten individuals who consume chicken at elevated levels. Legislation was proposed in 2009 in the USA Congress to ban roxarsone, and in June 2011, the Pfizer chemical company announced that they would no longer manufacture roxarsone in the United States. Nitarsone continued use as a poultry supplement until 2015, when it was removed from the market [7]. Arsenic supplements to poultry feed were never approved for use in the European Union, but they are extensively used today in China, where the allowable dose limit for chicken feed is currently 50 mg/kg [8,9].

The As contained in poultry feed is excreted from the animal in its original form [10]. Composting poultry litter degrades roxarsone to arsenate (AsO_4_^−^), which is a more toxic form of As that can be transported in water [10,11]. Additional roxarsone degradation products that have been observed in poultry litter include monomethylarsonic acid (CH_3_AsO(OH)_2_), dimethylarsinic acid ((CH_3_)_2_AsOH), and arsenite (AsO_3_^−^) [8]. Roxarsone has been shown to undergo biotransformation to inorganic arsenic in several studies [10,12,13,14]. Stolz et al. demonstrated the rapid biotransformation of roxarsone under anaerobic conditions by *Clostridium* species in chicken litter [11]. In addition to being water-soluble, and therefore transportable with groundwater, where it can enter the food chain, these arsenic substances are more toxic than roxarsone [15]. Roxarsone has also been shown to undergo photodegradation to form arsenate, nitrate, and additional breakdown products [16].

In 2011, typical roxarsone levels in commercial poultry feed were 30–50 mg/kg [17]. Elevated As levels in runoff water and soil pore water from fields treated with poultry litter are well documented [18,19,20,21,22,23,24,25,26]. Roxarsone is more strongly absorbed in some soils than in others [25]. The rate of biodegradation is greater in some soils than in others [27]. Adsorption onto mineral surfaces plays a role in the leaching rate for roxarsone from poultry litter and the breakdown product, arsenate. Soils that contain Fe and Mn release As more slowly because of the limited solubility of these complexes [28]. The ultimate fate of poultry litter As is to enter the vadose zone and be transported in solution from the field where the poultry litter was applied. The time required for As levels to return to pre-litter treatment values depends on the poultry litter application rate, the soil types, the mineral composition of the soil, and the available precipitation. 

Broiler feed contains supplements of Cu, Fe, Mn, Se, and Zn, which are added to ensure weight gain and minimize the health disorders in the poultry [29,30,31]. Unused trace metal supplements end up in the chicken manure, and different feed formulations result in changes to the metal levels in poultry litter [32]. Applications of poultry litter to agricultural fields then increase the concentrations of these metals in the soil. Long-term applications of poultry litter could potentially result in toxic levels of some metals [32,33]. 

Elevated levels of Cu and Zn in soils that have been treated with poultry litter have been reported by many investigators [29,34,35,36,37,38]. Cu and Zn are retained in the top 10 cm of soil that has been treated with poultry litter. Gupta and Charles reported a single study where soil Cu levels increased from poultry litter treatment, but the Zn levels were unchanged. Mn is another element that has been shown to build up in soil following poultry litter treatment, but not in all studies. Arsenic levels increase in soils that were treated with poultry litter containing roxarsone. Over time, As migrates down the soil column instead of remaining in the top 10 cm of the soil. Arsenic originating from poultry litter has been reported at a depth of 30 cm in two separate studies [37,39]. Differences in the trace metal content of soils that have been treated with poultry litter for extended periods of time are attributed to the rate at which poultry litter is applied to the soil and different formulations of the poultry feed [40,41].

A majority of the poultry farms in the Shenandoah Valley operate under contract to one of the large USA poultry producers. The farmers provide facilities and labor to raise either chickens or turkeys that are delivered to the farmers when the pullets are two days old. The poultry producer provides feedstock, and the formulations are proprietary. Farmers typically raise six flocks of small broiler chickens (1.8 kg/head) per year and apply the litter on fields, providing a nitrogen and phosphate source for crops. Nutrients that were added to the feedstock to enhance poultry growth (Cu, Mn, Zn, and As) end up in manure, and are then distributed on the fields used to grow corn, soybeans, and hay. The purpose of this study was to determine if the practice of applying poultry litter generated from chickens fed commercially formulated feedstock had a long-term effect on Cu, Fe, Mn, and Zn soil levels. A second objective was to determine if any arsenic remained in the soil five years after roxarsone was removed from the chicken feed.

## 2. Materials and Methods

### 2.1. Research Design

The farm that was chosen for this study is located in the Shenandoah River Valley of Rockingham County Virginia (38.5343, −78.7217). The Shenandoah Valley lies at the base of the Appalachian Mountains in the eastern United States. The study site consists of a fenced 2.1 ha pasture of bermudagrass (*Cynodon dactylon*), and the comparison site is an undisturbed woodland that has not been used for agricultural purposes. The study and comparison sites are located directly adjacent to each other, and the Rockingham County Soil Survey shows that the soil at both sites consists of Frederick and Lodi silt loams. The study and comparison sites are located in section 33C2 of the soil survey map [42]. The surface layer of Lodi soils consists of a dark grayish brown silt loam about 18 cm thick, and 1.3 m of subsoil that is brown and yellowish red silty clay loam and clay. The surface layer of Frederick soils is a 23-cm thick brown silt loam, with 1.5 m of subsoil that is a brown clay loam. Frederick and Lodi soils are mapped together, because they have no major differences [42]. 

The study site has been used as a pasture to graze cattle for the past 55 years. The comparison site, on the other hand, is an undisturbed woodland that has not been used for agricultural purposes. The study site was chemically killed in 1999 to eliminate invasive grasses and weeds, and sprigged with bermudagrass (*Cynodon dactylon*) using a no-till planter to prevent disturbing the surface. Bermuda and cool season grasses are the only crops that have been grown on the study site since 1999. The purchase of a calibrated manure spreader in 2000 made it possible to maintain accurate poultry litter application records. Poultry has been raised on this farm since 1979, starting with two flocks of range turkeys (20,000 head per flock). In 1980, broiler houses were built to house 36,000 broilers per flock. Six flocks were raised per year under contract with three major poultry integrators over 35 years in the poultry business. Chicken litter was applied to the study site annually and tested for macronutrients and Cu, Mn, and Zn. The dates of litter application and the Cu, Mn, and Zn concentrations of the chicken litter are listed in Table 1.

### 2.2. Field Sampling

Twelve soil samples were taken with a stainless steel soil auger [43] from both the study site and the comparison site on 6 February 2016, using a systematic, square grid sampling model [44]. Each square sampled represents approximately 0.18 ha. Each sample was split into a surface (top 10 cm) and a subsurface sample. The soil samples were put into ZipLock^©^ bags and placed on ice for transport to the laboratory. The soil samples were stored at 4 °C before analysis. Litter samples were taken from the floor of poultry houses that had been used to house the 2012 and 2014 flocks. These were the last chicken flocks produced at this farm, and the poultry houses had remained vacant and undisturbed from the time the chickens were harvested. Litter samples were put in ZipLock^©^ bags, placed on ice for transport, and stored at 4 °C.

### 2.3. Chemical Analysis

Extractable concentrations of Cu, Fe, Mn, Zn, and As in the soil samples were determined by atomic absorption spectroscopy. A soil sample of 3 g was added to 25 mL of Mehlich 3 Extraction Solution [45,46] and shaken at 200 rpm (revolutions per minute) at room temperature for six minutes. Each sample was then vacuum filtered to remove sediment and stored in polypropylene centrifuge tubes. Cu, Mn, Fe, and Zn concentrations were determined by flame atomic absorption spectroscopy (FAAS) with a Perkin-Elmer model 5100 FAA (Perkin-Elmer Corp., Norwalk, CT, USA) spectrometer and hollow cathode lamps [47]. Arsenic concentrations were measured with the same instrument, which was configured for graphite furnace atomic absorption spectroscopy [48]. An electrodeless discharge lamp (Perkin-Elmer Corp., Norwalk, CT, USA) was used for the arsenic analysis.

Cu, Fe, Mn, Zn, and As concentrations were determined in the poultry litter samples that were collected in February 2016, after digesting litter samples in a CEM Corporation MARS 5 microwave-assisted reaction system [49]. Litter samples were passed through a 3-mm soil sieve to remove large particles, and then dried overnight in a desiccator at room temperature. The dried litter samples were then homogenized by grinding approximately 5 g samples in a mortar and pestle. The homogenized litter sample was then passed through a 90-µm soil sieve before combining 100 mg of sample with trace metal grade HNO_3_, 5.0 mL, trace metal grade H_2_O_2_, 3.0 mL, and trace metal grade HF, 500 µL. The sealed reaction vessels were ramped from room temperature to 180 °C over a period of 5.5 min, and then held at 180 °C for an additional 9.5 min. Samples were filtered through a 0.2-µ polypropylene filter before use to remove fine particulates.

### 2.4. Statistical Analysis

Analysis of variance (ANOVA, α = 0.05) was performed with NCSS 9 Statistical Analysis and Graphical Software (Version 9.0.10, NCSS, LLC, Kaysville, UT, USA, 2013) [50]. The differences between groups were determined with Tukey’s multiple-difference test [51]. 

## 3. Results

### 3.1. Cu, Fe, Mn, Zn and As Application Rates

The poultry litter concentrations for Cu, Mn, and Zn in Table 1 represent snapshots of the trace metal content that was applied to the study site on that date, with data generated by established agricultural laboratories using standard analytical procedures. The values are in good agreement with previously reported literature values for trace metal content of poultry litter [52,53]. The median application rates average out the variances observed between the annual values, and give a good representation of the application rates of these metals over the course of 12 years. The Cu, Mn, and Zn concentrations in Table 1 were then used to calculate the application rates of these metals, as shown in Table 2.

Poultry litter consists of wood shavings, unconsumed pieces of feed, and dried poultry manure. The samples are very heterogeneous, leading to large standard deviations for the five metals tested, in spite of our efforts to homogenize the material and take representative samples. Litter samples taken from the broiler houses in 2016 were dried, homogenized with a mortar and pestle, and sieved through a 90-µm screen, and still gave answers with an average ±9.2% relative deviation. Sample homogeneity is one of the factors in the standard deviation of repeated analysis of the same sample. We repeated the analysis of the broiler house litter samples four times for each year, and obtained the results that are shown in Table 3. 

The data from the litter samples collected in February was used to calculate application rates for Fe and As, because the previous chemical analysis of litter samples did not include data for these elements. The Fe and As concentrations from the 2012 and 2014 poultry litter samples are shown in Table 4 along with application rates for Fe and As for 2012 and 2014.

### 3.2. Metal Concentrations from Soils

Twelve soil samples from both the study site and the comparison site were divided into surface and subsurface samples and treated with Mehlich extraction solution to measure the concentrations of Cu, Fe, Mn, Zn, and As that are available to plants. The extraction solutions were tested by atomic absorption spectroscopy to determine the *extractable* soil metal levels. The mean concentrations for Fe, Mn, Cu, Zn, and As are shown in Table 5.

**Figure 2:**
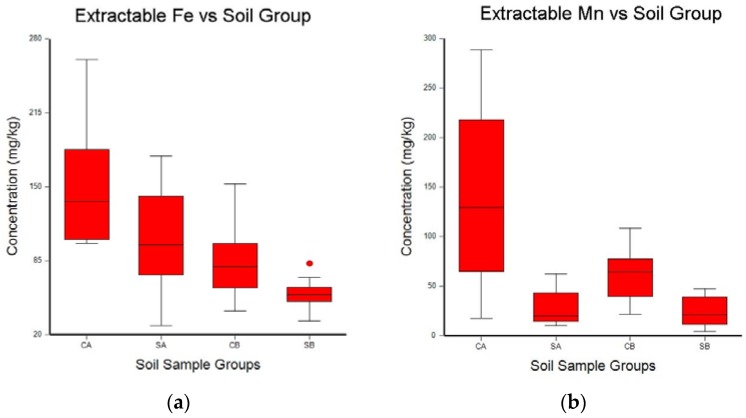
Box–whisker plots of the mean concentrations of extractable Fe (**a**) and Mn (**b**) in soil samples collected from the treated field (SA and SB) and the comparison site (CA and CB). Samples labeled A are from the top 10 cm of soil, and samples labeled B are from 10 cm to 20 cm below the surface. Whisker boundaries are set at the box edge ±1.5 times the interquartile range. Outlier boundaries are set at ±3.0 times the interquartile range.

**Figure 3:**
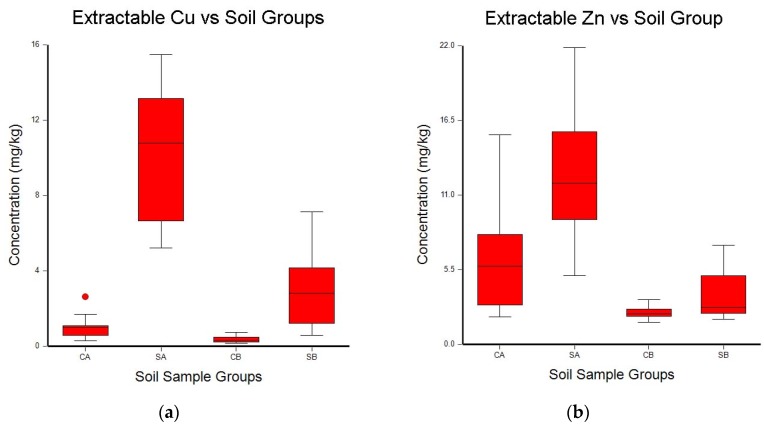
Box–whisker plots of the mean concentrations of extractable Cu (**a**) and Zn (**b**) in soil samples collected from the treated field (SA and SB) and the comparison site (CA and CB). Samples labeled A are from the top 10 cm of soil, and samples labeled B are from 10 cm to 20 cm below the surface. Whisker boundaries are set at the box edge ±1.5 times the interquartile range. Outlier boundaries are set at ±3.0 times the interquartile range.

**Figure 4:**
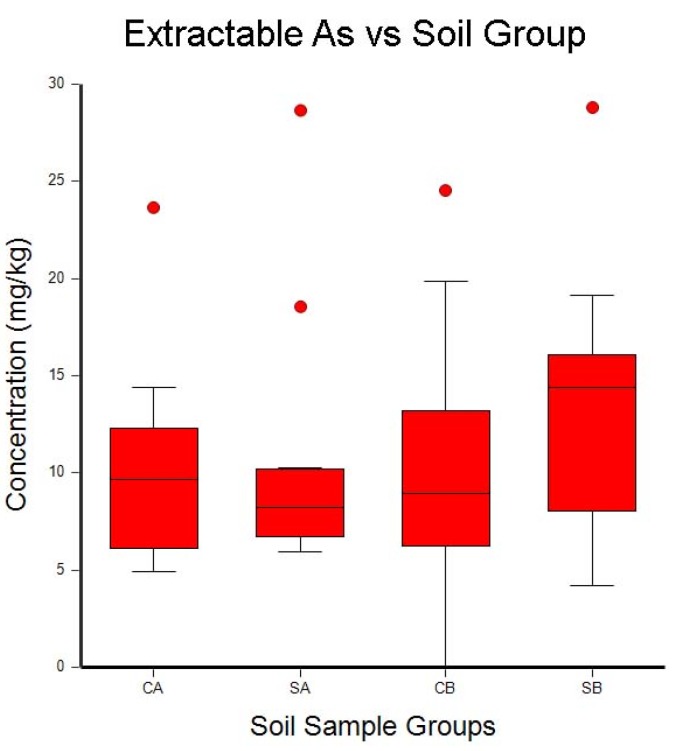
Box–whisker plots of the mean concentrations of extractable As in soil samples collected from the treated field (SA and SB) and the comparison site (CA and CB). Samples labeled A are from the top 10 cm of soil, and samples labeled B are from 10 cm to 20 cm below the surface. Whisker boundaries are set at the box edge ±1.5 times the interquartile range. Outlier boundaries are set at ±3.0 times the interquartile range.

A one-way ANOVA analysis was used to determine the differences between surface and subsurface samples, and between the study site and the comparison site. Box–whisker plots of the ANOVA results are shown in Figure 2, Figure 3 and Figure 4. The box–whisker plots for the soil metal results clearly show the differences that were observed for these five metals between the study site and the comparison site, and between the surface samples and the subsurface samples.

## 4. Discussion

### 4.1. Arsenic

Extractable As concentrations were unchanged between the surface and subsurface samples, and they were also unchanged between the study site and the comparison site. This result is not surprising, because the soil samples in this study were taken approximately two years after the last treatment with poultry litter containing roxarsone. Previous studies have shown that roxarsone is transformed to arsenate in the soil and removed from the treated fields through leaching with soil water [25]. The As leaching rates do not follow a straightforward kinetic process, and the biotransformation of roxarsone is thought to contribute to the loss of arsenic in fields treated with poultry litter [23]. These results show that soil arsenic levels, five years after the last application of poultry litter containing arsenic, are at background arsenic levels for these soils.

### 4.2. Micronutrients

Cu, Fe, Mn, and Zn are essential micronutrients that are frequently added to fertilizers to supplement natural soil levels and increase crop yield [26,29,54]. At elevated levels, these trace metals become toxic [55], inhibiting plant growth. Toxic metals may (1) cause oxidative stress in the plant’s metabolism system, (2) alter the anatomy of leaves roots and stems, resulting in a decreased uptake and translocation of essential nutrients, or (3) inhibit karyokinesis and cytokinesis [56]. Phytotoxicity varies between plant species [57], and is affected by soil composition, other trace metals that may be present in the soil [58], and soil pH [54,59]. Table 6 lists the critical deficiency level and critical toxicity level for Cu, Fe, Mn, Zn, and As.

### 4.3. Iron and Manganese

Fe and Mn exhibited similar behavior in this study. Extractable Fe and Mn concentrations were higher in surface samples than in subsurface samples. Also, extractable Fe and Mn concentrations were higher in the comparison site. Over the course of this experiment, both Fe and Mn were depleted from the soil at the study site. Bermudagrass (*Cynodon dactylon*) has been shown in several studies to lower soil Fe and Mn concentrations [66,67]. Provin et al. showed that the dynamics of Mn, Fe, Cu, and Zn removal from soil by bermudagrass was seasonal and related to dissolved organic carbon and precipitation [68].

### 4.4. Copper and Zinc

Cu and Zn exhibited similar behavior in this study. However, the trends over time for extractable Cu and Zn were opposite to the trends observed for Fe and Mn. Cu and Zn levels were higher in both the surface and subsurface samples for the study site at the end of the experiment. Also, extractable Cu and Zn concentrations were significantly higher in the treated field, for both surface and subsurface samples, over the levels observed for the comparison site. These findings agree with the majority of previous studies of Cu and Zn soil levels following treatment with poultry litter. In a typical study of manure-amended fields, Brock et al. analyzed soil and leachate water samples taken from 109 fields in southern New York, USA, for Cu and Zn. The fields had been treated with liquid dairy manure and solid poultry litter for 40 years. The authors of this study concluded that although Cu and Zn soil levels increased as a result of the dairy manure and poultry litter treatments, the soil Cu and Zn concentrations had not reached toxic levels. They also concluded the greatest environmental threat from treating the soil with manure and poultry litter was phosphate runoff, rather than toxic metal buildup. Novak et al. reported Cu and Zn accumulation in soil following long-term applications of swine manure to a field planted with bermudagrass (*Cynodon dactylon*) [69].

## 5. Conclusions

Continuing to manage the pasture with an annual application of poultry litter will result in changes to the four essential micronutrients studied in this project. Fe and Mn are depleted from the soil, while Cu and Zn levels are increasing, and although roxarsone was present in the poultry litter applied from 1999 to 2012, current As concentrations are at background levels. It is unlikely that continuing to apply poultry litter at the rates reported in this study will increase soil metal concentrations to the critical toxic level for the four metals studied in this project (Cu, Fe, Mn, and Zn).

## Figures and Tables

**Table 1:** Poultry litter applications to the study site, showing Cu, Mn, and Zn concentrations in the litter. Concentrations expressed as mg/kg.

Date	Cu	Mn	Zn	Analyst
1 March 1999	704	293	409	Maryland Cooperative Extension Service
10 February 2000	789	491	418	Maryland Cooperative Extension Service
4 March 2004	721	552	465	Clemson University Extension Service
18 February 2005	674	615	457	Clemson University Extension Service
28 February 2007	391	579	469	Clemson University Extension Service
16 May 2007	391	614	489	Clemson University Extension Service
22 May 2008	316	511	404	Clemson University Extension Service
28 August 2009	373	560	363	Clemson University Extension Service
22 April 2010	306	517	355	Clemson University Extension Service
1 September 2010	222	482	349	Clemson University Extension Service
22 August 2012	287	375	331	Clemson University Extension Service
6 August 2014	450	831	674	Clemson University Extension Service
Median Values	391	535	414	
Std. Deviation	188	189	113	

**Table 2:** Application rates of Cu, Mn, and Zn to the study site. Application rates are expressed as kg/ha.

Date	Cu	Mn	Zn
1 March 1999	2.37	0.99	1.38
10 Febuary 2000	2.66	1.65	1.41
4 March 2004	2.43	1.86	1.57
18 Febuary 2005	2.27	2.07	1.54
28 Febuary 2007	1.32	1.95	1.58
16 May 2007	1.32	2.07	1.65
22 May 2008	1.06	1.72	1.36
28 August 2009	1.26	1.89	1.22
22 April 2010	1.03	1.74	1.20
1 September 2010	0.75	1.62	1.18
22 August 2012	0.97	1.26	1.12
6 August 2014	1.52	2.80	2.27
Median Values	1.32	1.80	1.39
Std. Deviation	0.61	0.61	0.37

**Table 3:** Cu, Fe, Mn, and Zn concentrations in poultry litter samples taken in February 2016. The results are reported for four separate analyses of each litter sample. The large standard deviations reflect the sample homogeneity problems associated with analyzing poultry litter.

Sample	Cu	Fe	Mn	Zn
Litter from 2012	281 ± 13.4	1106 ± 296	1032 ± 71	687 ± 22
Litter from 2014	441 ± 38	2200 ± 104	788 ± 65	570 ± 60

**Table 4:** Iron and arsenic concentrations with standard deviations from poultry litter samples. Concentrations are an average of four analyses from each litter sample, and are expressed as mg/kg. The application rates are expressed as kg/ha.

Sample	Litter Concentrations		Application Rates		Analyst
	Fe	As	Fe	As	
Litter from 2012	2200 ± 252	2.5 ± 3.0	7.41	0.009	This study
Litter from 2014	1106 ± 89	5.2 ± 0.4	3.73	0.013	This study

**Table 5:** Mean concentrations of extractable Fe, Mn, Cu, Zn, and As in soils. Concentrations are expressed as mg/kg.

Soil Sample	Fe	Mn	Cu	Zn	As
Treated—Surface	104 ± 44	29 ± 19	10.3 ± 3.5	12.3 ± 4.6	10.5 ± 6.6
Treated—Subsurface	56 ± 13	24 ± 15	2.9 ± 1.9	3.6 ± 1.8	13.6 ± 6.6
Comparison—Surface	149 ± 50	138 ± 87	1.0 ± 0.6	6.3 ± 3.9	10.4 ± 5.1
Comparison—Subsurface	83 ± 31	63 ± 27	0.35 ± 0.2	2.3 ± 0.5	10.3 ± 6.6

**Table 6:** Critical deficiency levels and critical toxicity levels for As, Cu, Fe, Mn, and Zn in soils.

Element.	Critical Deficiency Level (mg/kg)	Critical Toxicity Level (mg/kg)	Reference
Cu	1–5	20–30	[60]
Fe	3–5	680–850	[61,62,63]
Mn	10–20	200–3500	[60]
Zn	0.58, 1.23, 1.35	100–300	[60,64,65]
As	Not Required	<2–80	[60]
